# Understanding User Reactions and Interactions With an Internet-Based Intervention for Tinnitus Self-Management: Mixed-Methods Process Evaluation Protocol

**DOI:** 10.2196/resprot.5008

**Published:** 2016-03-23

**Authors:** Kate Greenwell, Magdalena Sereda, Neil Coulson, Derek J Hoare

**Affiliations:** ^1^ National Institute for Health Research (NIHR) Nottingham Hearing Biomedical Research Unit Nottingham United Kingdom; ^2^ Otology and Hearing Group, Division of Clinicial Neuroscience School of Medicine University of Nottingham Nottingham United Kingdom; ^3^ Division of Rehabilitation and Aging School of Medicine University of Nottingham Nottingham United Kingdom

**Keywords:** Tinnitus, health, internet, program acceptability

## Abstract

**Background:**

Tinnitus is a common medical symptom that can affect an individual’s emotional and functional quality of life. Psychological therapies are acknowledged as beneficial to people with tinnitus; however, such therapies are not always readily accessible. With their global reach, automated Internet-based interventions have the potential to reduce the disparity in access to psychological support that people with tinnitus currently experience. However, the evidence on the acceptability and efficacy of these interventions is lacking. Process evaluations that develop an in-depth understanding of how users experience these interventions provide an essential first step when evaluating complex psychological interventions.

**Objective:**

To describe the protocol for a study that will explore past, current, and new users’ reactions to and interactions with the Tinnitus E-Programme, an Internet-based intervention for the self-management of tinnitus.

**Methods:**

Two parallel mixed-methods studies will be carried out with 2 different populations. Study 1 will use an online survey to gather past and current users’ views of the program. Study 2 will recruit new program users to take part in an interview and complete a relaxation log to explore how well they were able to implement the skills they learned during the program in their everyday lives. The findings from both studies will be triangulated to develop an in-depth understanding of the program’s mechanisms of impact and identify any implementation or contextual factors that strengthen or impede its delivery and functioning.

**Results:**

Study 1 is open for recruitment with a projected completion in June 2016 and Study 2 was completed November 2015. At the time of submission, 36 participants have been recruited to Study 1 and 12 participants have taken part in Study 2.

**Conclusions:**

Findings will inform the optimization of the Tinnitus E-Programme and guide future evaluation work to assess the program’s effectiveness as a therapy for people with tinnitus.

## Introduction

### Background

Tinnitus (ringing in the ears) affects approximately 10%-18% of the population [1-3] and is characterized by a conscious perception of a sound without a corresponding external source. Tinnitus can significantly affect an individual’s quality of life, leading to emotional strain (eg, irritation, depression, frustration, anxiety), sleep disturbances, concentration difficulties, and disruptions to social and work life [4-6].

In the absence of a definitive biomedical cure, current health care strategies focus on supporting people to manage their tinnitus to ultimately reduce the tinnitus percept and associated psychological distress [7]. Currently, access to psychological therapies, such as cognitive behavior therapy, for people with tinnitus is limited [8-10], with such specialist psychological services generally being reserved for those with the greatest need [7]. Internet-based interventions, with their global reach, have the potential to reduce this disparity and improve access to psychological support for people with tinnitus. They also provide an alternative for those unable or unwilling to access traditional face-to-face psychological services [11,12].

There is evidence to suggest that Internet-based interventions are effective for reducing tinnitus distress and psychological comorbidity while improving quality of life [13-16]. However, the current evidence-base has focused on therapist-guided interventions, which lack the scalability necessary for equitable access. On the other hand, the evidence-base for unguided (or automated) Internet-based interventions is limited and less clear [14,17,18]. One such example is the Tinnitus E-Programme, an Internet-based intervention to support tinnitus self-management that was developed in the United Kingdom [19]. The program comprises several self-management components including: education about tinnitus and its management; information about available resources; training in psychological strategies (ie, relaxation, cognitive restructuring); peer support via an online discussion forum; and self-monitoring of tinnitus outcomes. Its multicomponent nature defines the program as a complex intervention [20]. Although freely available online, we currently know little about how the Tinnitus E-Programme is used, how it works, the circumstances in which it works best, and whom it works best for.

To evaluate the Tinnitus E-Programme, we are guided by the Medical Research Council’s guidance on developing and evaluating complex interventions [20] that emphasizes the importance of carrying out adequate pilot and feasibility work prior to a definitive randomized controlled trial. Interventions should be tested using a phased approach whereby a series of pilot and exploratory studies address any key uncertainties in the intervention design. Development and evaluation stages are iterative, with researchers moving back and forth between each stage. Any intervention modifications and future evaluation work is thus informed by an evolving evidence-base produced by these pilot studies. Without adequate development and piloting work, interventions are likely to be weaker and difficult to evaluate [20].

A useful first step when evaluating developed interventions is to carry out a process evaluation. This can provide information on the (1) implementation, (2) mechanisms of impact, and (3) contextual factors that influence the delivery and outcome of the intervention [21]:

1. Implementation is concerned with what is delivered in practice and the structures and resources required for successful implementation [21] and is typically conceptualized in terms of fidelity (ie, was the intervention developed and used as intended?), dose (ie, how much of the intervention was delivered and received?), reach (ie, to what extent did the intervention reach its target audience?), and enactment (ie, to what extent was the knowledge or skills participants acquired during the intervention applied to everyday life?) [22-24]. In the context of Internet interventions, usability testing is essential for ensuring that the intervention performs as intended and identifying and eliminating any barriers to easy and effective use by its target population [25]. Exploring intervention usage or attrition can also provide useful implementation insights [26].
2. Mechanisms of impact is concerned with how the intervention components—and a user’s interactions with them—lead to the desired changes in outcome [21]. That is, what are the mechanisms through which Internet interventions work (ie, how they work) and the factors that are essential for their success (ie, what makes them work)? Qualitative methods can be particularly helpful for exploring relatively unknown mechanisms of impact and allow unintended and/or unanticipated intervention consequences to be explored [27]. This may include identifying negative intervention outcomes or benefits not initially anticipated by the intervention developers or evaluators. For example, in a mixed-methods evaluation of psychological therapies for multiple sclerosis, Dennison et al’s [28] qualitative interview findings uncovered a disparity between participants’ perceptions of what the therapy changes were and the predetermined outcomes measured in the parallel efficacy trial.
3. Context is concerned with how external factors may strengthen or impede the delivery and functioning of the intervention [21]. Such external factors may include preexisting circumstances, skills, resources, and attitudes of the target population. A thorough understanding of the intervention context is helpful for explaining any variability in intervention outcomes [28,29].

This study will carry out a process evaluation of the Tinnitus E-Programme to further our understanding of the program’s mechanisms of impact and identify any implementation or contextual factors that strengthen or impede its delivery and functioning. Most process evaluations have been carried out on people who were recruited offline and are using the intervention for the first time as part of a research study. This reduces the findings’ external validity and relevance to real-world practice [30-32]. This study will recruit users of the live program, as well as people with tinnitus who have not used the program previously. Mixed methods will be used to develop an in-depth understanding of the perspective of the target user [33]. The findings will inform the optimization and future evaluations of the program, as well as the development of other similar internet-based self-management interventions.

### Aims

To explore past, current, and new users’ reactions to and interactions with the Tinnitus E-Programme. The specific aims are to explore:

1. The acceptability and usability of the program (implementation, context);
2. How users engage with the program (implementation, mechanisms of impact, context);
3. Users’ perceptions of the processes and outcomes of the program (mechanisms of impact, context);
4. User enactment of the relaxation skills learned in the program (implementation, mechanisms of impact, context).

## Methods

### The Intervention

The Tinnitus E-Programme [34] is a 10-week Internet-based self-management intervention for tinnitus. It was developed by a hearing therapist/psychotherapist in private practice and was launched in 2009. It is live online and free to access without registration. The website currently receives approximately 1000 visits per month. The program includes: (1) downloadable information resources to provide education about tinnitus and its management; (2) training/rehearsal for psychological strategies, including relaxation and brief cognitive restructuring skills training; (3) online discussion forum to provide social support from peers and lay and professional moderators; (4) self-monitoring of tinnitus distress using the Tinnitus Handicap Inventory [35]; and (5) information about available resources, including book references and hyperlinks to other websites or services. Educational topics covered by the information resources include the mechanisms of tinnitus, stress and its management, attention focus, and negative thinking. Several behavior change techniques are also used to promote relaxation behavior (eg, goal setting, action planning, behavioral practice/rehearsal). Further information about the Tinnitus E-Programme’s specific components, techniques, and mode of delivery can be found elsewhere [19].

Program content is delivered across 6 weekly modules, followed by a 4-week maintenance period where users are asked to continue the daily relaxation goals set in the previous period. No additional intervention content or support is delivered during this maintenance period. A recommended program structure is given; however, users have free choice regarding which components they access and in what order they access them. The express aim of the program is to reduce tinnitus distress, but the precise mechanisms by which this change should occur are not yet established.

### Paradigm and Design

This research will adopt pragmatism [36] as its overarching methodological paradigm. Pragmatism is primarily concerned with the consequences of research. Unlike other paradigms, such as postpositivism and constructivism, pragmatism is not tied to one particular epistemology or data collection method (ie, qualitative or quantitative). Rather, methods are chosen based on “what works,” that is, their ability to successfully answer a particular research question.

Consistent with this approach, 2 parallel mixed-methods studies will be carried out with 2 different populations to evaluate the program from multiple perspectives. This design will allow triangulation of research data and methods that will generate and compare complementary perspectives and contexts. The intention is that the use of both qualitative and quantitative research methods and more than 1 study population will provide a more complete, in-depth, and valid understanding of the phenomenon than if only 1 method or population was used [36,37]. Mixed methods have been used successfully for process evaluations [21] and evaluations of digital interventions [26,38].

Study 1 will explore how past and current users react to and interact with the program in the real-world, outside of a research context. Due to technical limitations of the program, it is not possible to monitor actual program usage. Therefore, an online survey will be used to gain self-reports of how users interacted with the program, as well as users’ reactions to the program. A convergent mixed-methods design [36] will be used in which qualitative and quantitative methods are implemented simultaneously and given equal weight, but the data will be analyzed separately. The online survey will use open (ie, qualitative) and closed (ie, quantitative) questions to elicit users’ views. Specifically, a data-validation variant of this mixed-methods design will be used [36] in which the qualitative data is used to validate and elaborate on the quantitative data.

Study 2 will recruit a cohort of individuals with tinnitus who have not previously used the Tinnitus E-Programme in an attempt to gather more in-depth, timely, and diverse views and experiences. Participants will complete the program for the first time and take part in a semistructured interview. Participants will complete a relaxation log to explore the extent to which they enacted the relaxation skills learned in the program and any barriers to doing so. An adapted version of an embedded mixed-methods design will be used [36] in which both the qualitative and quantitative relaxation log data collection and analysis is embedded within an overall qualitative research design. As such, the relaxation log data will be secondary to the qualitative data and will be used to enhance understanding of the qualitative interview findings.

The findings of the 2 mixed-methods studies will be triangulated in an overall interpretation. This research has ethical approval from the University of Nottingham Research Ethics Committee (Reference Number: Q11122014 SoM NIHR RHA QEST).

### Study 1: Online Survey With Current and Past Users

#### Participants

Participants in Study 1 will self-select, based on their own judgements of whether they meet the following inclusion criteria: (1) adults aged 18 years and over, (2) ability to read English, (3) access and ability to use the Internet, and (4) have visited the Tinnitus E-Programme website or used the program. Participants may have accessed the program anytime over the last 6 years since the program was launched. There will be no exclusions regarding length of time since starting the program in order to maximize recruitment for this very specific population. The program does not specify any inclusion criteria, as the intention is that it is suitable for everyone with tinnitus. In keeping with this, there are no exclusions regarding tinnitus duration, severity, or co-morbidities in an attempt to recruit all potential users.

#### Recruitment

Past and current program users will be invited to take part in an online survey hosted on SurveyMonkey. Advertisements will be posted on the Tinnitus E-Programme website and online discussion forum, along with the participant information sheet. The survey will also be advertised via social media and national charities in an attempt to reach those who no longer interact with the program or website. Email invitations and the participant information sheet will also be sent to those who registered with the program website or online discussion forum. Sample sizes for similar descriptive online survey studies have been between 50-249 individuals [39-42]; therefore, a sample size of above 50 will be deemed acceptable. The survey will be closed after 3 months or until at least 50 participants have been recruited.

#### Online Survey: Development and Piloting

The initial survey design was informed by the study rationale, relevant literature, and the comprehensive intervention description developed previously [19]. The survey focused on the information resources (ie, education about condition and management, information about available resources, training/rehearsal for psychological strategies), relaxation exercises (ie, training/rehearsal for psychological strategies), Tinnitus Handicap Inventory (ie, self-monitoring of condition), and online discussion forum (ie, social support).

The survey uses a mix of closed and open questions concerned with: (1) reasons for participating or not participating in the program, (2) how the program was used, (3) usability of the program, (4) acceptability of the individual program components, and (5) benefits derived from the program and its impact on tinnitus management. Demographic data will also be collected on gender, age, country of residence, whether English is their first language, presence of tinnitus, tinnitus duration, and tinnitus management strategies used previously or currently.

To assess the acceptability and face validity of the survey, an initial set of survey questions were reviewed by a public and patient involvement (PPI) panel assembled for the purposes of this study. The panel included 4 people with tinnitus and/or hearing loss who were recruited from an established National Institute for Health Research (NIHR) Nottingham Hearing Biomedical Research Unit (NHBRU) PPI panel and 1 voluntary sector representative from the British Tinnitus Association who had experience in writing communication materials for people with tinnitus. Panel members were chosen from a wider established PPI panel, based on their availability and previous experience of reviewing research materials. A focus group was carried out with the PPI panel to gather initial feedback on a paper version of the draft survey. The focus group was attended by the first author and co-facilitated by the PPI manager at NHBRU and an external facilitator who was not involved in the study but was familiar with issues relevant to hearing research. Panel feedback focused on the relevance and ordering of the questions, language used, and appropriateness of question type (ie, closed or open). Following the focus group, the survey was uploaded onto SurveyMonkey, and this online version was circulated to the PPI panel via email for additional comments. The panel was satisfied with the online version and no further amendments were made.

The final online survey was subsequently piloted with 3 Tinnitus E-Programme users recruited from the program’s online discussion forum. These participants completed the online survey and answered 4 additional questions about the survey length and relevance of the questions and closed-question answers. Informed consent was gained from these pilot participants who were told that their answers may or may not be used in the final analysis, depending on the outcome of the pilot.

All 3 participants reported that the survey took less than 30 minutes and “just the right amount of time” to complete. One participant suggested that it would be helpful to add a free-text comments box next to some of the closed questions to allow people to clarify their answers. The same participant also suggested adding a “cannot remember” option for the questions regarding program usage (eg, did you use the online discussion forum?) for those who used the program a long time ago. Both of these changes were made to the final survey. As these amendments were minor, the pilot data was retained for inclusion with the main study. A copy of the final survey can be found in Multimedia Appendix 1.

The online survey is anonymous to encourage participation and only 1 submission per computer will be allowed. Participants will be given a 14-day period in which they can request to have their answers deleted. After this period, their answers will be downloaded onto university servers and cannot be deleted. Participants will be asked to provide a security word as part of the survey and will be asked to recite this for data identification purposes should they wish to withdraw their data from the study.

#### Analysis

Answers to closed questions will be analyzed in IBM’s SPSS Statistics 22 using descriptive statistics, including frequencies and percentages, and each statistic carried out on complete data only. Answers to the open questions will be analyzed separately using inductive thematic analysis [43] and analysis informed by guidelines for establishing validity in qualitative research [44,45]. QSR’s NVivo 10 qualitative data analysis software will be used to provide an audit trail.

First, the 3 coders (KG, MS, DH) will familiarize themselves with the data through repeated reading of the survey answers. Second, KG will utilize line-by-line coding, a technique from grounded theory [46], in which each line of your transcript is coded. This ensures the coder remains open to the data and that subtle nuances in it are not missed. Codes will be kept close to the text and participants’ own language will be used wherever possible. KG will develop a coding manual that will list all codes, including descriptions and example quotes from the text [47]. The coding manual will improve the rigor of the research while also providing an audit trail for analysis decisions.

Third, at least 1 other coder (MS, DH) will independently apply the coding manual to all transcripts to clarify ambiguous codes, remove duplicate codes, and identify data that did not fit the coding scheme. Coding will be compared and discussed between coders and subsequent modifications made to the coding manual. Fourth, coders will collectively organize these codes into overarching themes and the coding manual will be updated accordingly. The constant comparison method [48], a grounded theory technique, will be used to compare codes across different participants, contexts, and situations. Disconfirming case analysis [45] will be used to actively identify data that does not fit with the identified themes. The final interpretations will be reviewed and agreed by all authors. Participant quotes will be used in the final write-up to illustrate the themes.

Consistent with a data-validation variant of the convergent mixed-methods design [36], the qualitative findings will be used to validate and elaborate on the quantitative data.

### Study 2: Interviews and Relaxation Log With New Users

#### Participants

Participants in Study 2 will self-select, based on their judgement of whether they meet the following inclusion criteria: (1) adults aged 18 years and over, (2) ability to read English, (3) access and ability to use the Internet, (4) have self-reported tinnitus, (5) reside in the United Kingdom, and (6) have not previously used the Tinnitus E-Programme. Again, as the program is meant to be suitable for all tinnitus users, participants were not excluded based on any tinnitus-related characteristics.

#### Recruitment and Procedure

A purposive sample of people will be chosen from the NHBRU research database, which includes approximately 900 UK residents with tinnitus who have agreed to be contacted about research. Maximum variation sampling [49] will be used to ensure that a diverse sample with different demographics (eg, gender, age) is chosen. As recruitment progresses, targeting will become more specific as participants with certain characteristics (eg, hearing loss, short tinnitus duration, younger age) are actively sought out to fill any demographic gaps in the current sample. An email invitation, together with a participant information sheet, will be sent to selected database members by a member of the research team.

**Figure 1:**
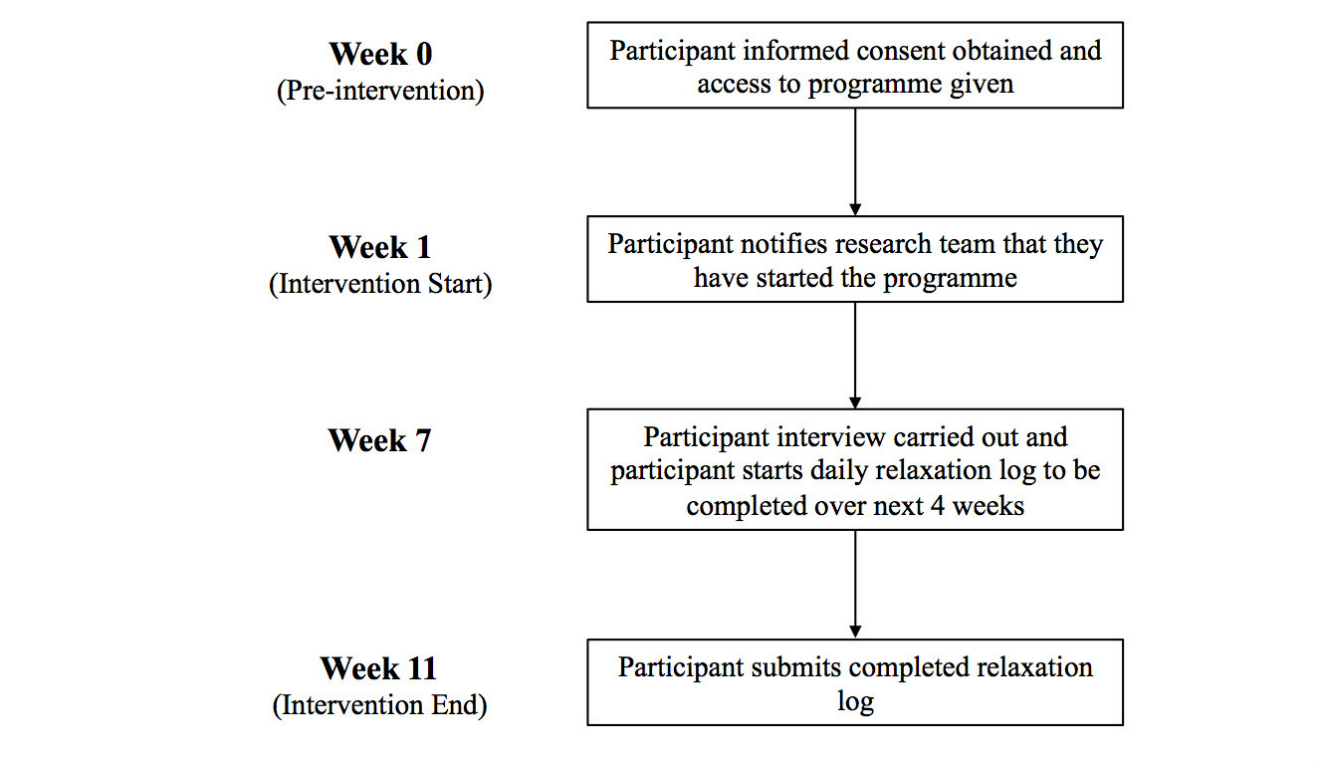
A flow chart showing the procedure for Study 2.

The procedure for Study 2 is illustrated in Figure 1. Once participants have expressed an interest in the research, the researcher will gain their informed consent using a paper or electronic consent form. Recruited participants will then be sent the hyperlink to the Tinnitus E-Programme and asked to notify the researcher once they start using the program. An interview will be organized for approximately 6 weeks after their start date. During this time, participants should have sufficient time to complete the first 6 sections of the program and be progressing into the maintenance phase. One week before their interview date, participants will be emailed a set of sample interview questions, a hyperlink to their online relaxation log, and instructions on how to complete the log. Participants were sent a set of sample interview questions to encourage transparency with the interview process and improve recall by giving participants time to think about the different topic areas and revisit the website if needed [50].

Interviews will be held no later than 8 weeks after the participant’s start date to explore how acceptable the 6-week timeline is and to ensure maximum recall of intervention experiences. Participants will be asked to complete a daily relaxation log on paper or online over the following 4 weeks. Email reminders will be sent to those who have not yet started the program, organized an interview date, or completed their relaxation log. Recruitment will cease once data saturation has been reached for the interviews; that is, when no new themes are emerging [51].

#### Interviews

An interview guide was developed that was informed by the literature, intervention coding, and study rationale. Specifically, the interview questions are concerned with how people used the program; reasons for any nonusage; experiences of using the program; expectations of the program; usability and acceptability of the program, as well as its individual components; benefits derived from the program; and suggested improvements to the program. The interview guide can be found in Multimedia Appendix 2. The interview guide was reviewed by the NHBRU PPI panel and piloted with a previous user of the Tinnitus E-Programme. No modifications resulted from this process.

Demographic data—including gender, age, ethnicity, and tinnitus duration—will also be obtained. Interviews will be carried out by the first author, a health psychologist and PhD student experienced in qualitative interviewing who was not involved in the Tinnitus E-Programme’s development. Interviews will last no longer than 1 hour. Participants will be given the choice of being interviewed in person at the research unit, over the phone, or via video chat. In an attempt to be inclusive, those with severe or profound hearing loss will also be offered the option to be interviewed using text communication methods (eg, instant messaging or email). The audio from the interviews will be recorded using a digital voice recorder and transcribed verbatim. The text from the textual communication methods will be saved electronically.

#### Relaxation Log

The relaxation log will assess users’ enactment of the relaxation goals set by the program during the 4-week maintenance period. An online relaxation log will be created for each participant using Google Sheets, an Internet-based spreadsheet program. Google Sheets will be hosted on the NIHR Google Hub, a secure online file storage system. The relaxation log is in tabular format with 4 columns and 28 rows representing each day of the 4-week period. Users will be required to answer the following 3 questions each day:

1. Did you practice the mind calming breathing exercise 3 or more times today?
2. Did you practice any of the 30-minute relaxation exercises today?
3. Did you use any other parts of the Tinnitus E-Programme today? If yes, please write which parts. If no, please write “no.”

There is also a free-text comments box to write any other comments for each day. At the end of week 10, participants will be asked to answer 1 final open question: “Did you practice the recommended relaxation exercises every day? If not, could you tell us about some of the things that made it difficult to do so?”

Participants will have the option of either accessing and completing their online log each day or printing and completing a paper copy. The completed paper copy may be posted or transferred onto the online log. Participants will be provided with instructions for completing their online relaxation log. Relaxation logs will be anonymous, identified only by a unique participant identification code. Each participant will be given a unique hyperlink to access their personal log, and only the participant and researcher will have access to this hyperlink.

#### Analysis

The interview data and open-question responses from the relaxation logs will be analyzed together using the same inductive thematic analysis strategy outlined in Study 1. The line-by-line coding will begin during data collection to help the interviewer to reflect and learn from previous interviews and refocus future interviews [46]. The quantitative relaxation log data will be analyzed using frequencies and percentages, including complete data only. This quantitative data will provide a secondary and supportive role to the qualitative data and will be used to enhance the qualitative accounts.

### Overall Interpretation

The findings from the 2 mixed-methods studies will be triangulated (ie, compared and contrasted) at the discussion-writing stage to produce an in-depth understanding of the program’s mechanisms of impact and identify any implementation or contextual factors that strengthen or impede its delivery and functioning. Triangulation will allow the findings from each study to be corroborated and validated [36,37].

## Results

At the time of manuscript submission, 36 participants have consented to take part in the online survey in Study 1. Thirty of these participants went on to answer questions about the program. For Study 2, 12 participant interviews have been completed and 6 relaxation logs submitted. Data collection for Study 2 was completed November 2015. Study 1 is open for recruitment and data collection will complete in June 2016.

## Discussion

This protocol describes 2 mixed-methods studies to evaluate the Tinnitus E-Programme, an Internet-based intervention for tinnitus self-management. A process evaluation will explore past, current, and new users’ reactions to and interactions with the program.

Ultimately, the findings of this research will provide the missing evidence-base that is necessary to guide future optimization and evaluation work for the program. First, the identification of any implementation or contextual factors that impede the delivery and function of the program will help us to decide which amendments need to be made to improve the program’s content, usability, and enactment for future users. Second, an in-depth understanding of the psychosocial context in which people with tinnitus interact with the program will provide insight into the circumstances in which the program works best and who is likely to benefit most from it. This can help guide decisions regarding appropriate research conditions and inclusion criteria for future evaluation studies. Third, understanding users’ perceptions of the outcomes of the program can guide evaluation choices regarding appropriate outcome measures. Finally, understanding the program’s mechanisms of impact can give us an understanding of how the program works and what makes it work. Such an understanding has wider implications for the management of tinnitus and can also inform the development of other Internet-based programs for people with similar conditions.

### Limitations

This research has some limitations or challenges that need to be considered. As registration to the program is not mandatory and users can choose to complete it anonymously, we have no way of knowing how many people have previously used, or are currently using, the live program. This makes it difficult to reliably estimate the sample size and accurately assess external validity for Study 1. It is also not possible to track past and current users who did not register, making this target population potentially hard to reach and recruit. This limitation also means that convenience sampling was the only feasible sampling method, which may introduce a self-selection bias. The current program does not monitor actual program usage, which means that it will not be possible to validate whether participants actually used the program and their self-reported usage. However, the focus of this exploratory study is on the participants’ accounts of their usage and reasons for usage or any nonusage.

Participants recruited to Study 1 are likely to represent a particularly motivated and satisfied group of users. Those who chose not to use the program, gained no benefit from the program, or no longer use the program are less likely to take part. Study 2 will provide more diversity as it will introduce a group of people to the dataset with different motivations (eg, to support tinnitus research, looking to benefit from a novel intervention). Once recruited, Study 2 participants will be encouraged to continue onto the interview, even if they did not complete or benefit from the program.

In Study 1, there were no exclusions regarding length of time since completing the program to maximize recruitment for this very specific population. Some of the participants may have completed the program as long as 6 years ago, which may introduce a recall bias.

### Conclusions

There are also several strengths of this research. First, the proposed evaluation is being carried out by an independent research team who were not involved in the development of the program. This will minimize any biases that might be present during data collection, analysis, and interpretation. Second, 2 different populations—current or past and new program users—will be studied, allowing us to evaluate the program from 2 different but complementary perspectives and contexts. Combined with the use of mixed methods, this design will provide a more complete, in-depth, and valid understanding of users’ reactions to and interactions with the program. Finally, this study will explore users’ enactment of the relaxation skills learned in the program. This aspect of intervention implementation is more commonly explored in behavior change research where integrating new actions into everyday life is the ultimate outcome of interventions [52]. Enactment has rarely been studied in research on interventions addressing psychosocial outcomes [22], with most research focusing on dropout or nonusage attrition [17,53,54]. This evaluation will also use mixed methods to relate individual assessments of enactment with user’s qualitative accounts of their experiences and reactions to this skills training.

## Appendix

Multimedia Appendix 1 Online survey for Study 1.

Multimedia Appendix 2 Interview guide for Study 2.

## Abbreviations

NHBRU: Nottingham Hearing Biomedical Research Unit
NIHR: National Institute for Health Research
PPI: public and patient involvement

## References

[1] Davis A, El Refaie A, Tyler RS (2000). Epidemiology of Tinnitus. Tinnitus Handbook. 1st ed.

[2] Park B, Choi HG, Lee H, An S, Kim SW, Lee JS, Hong SK, Kim H (2014). Analysis of the prevalence of and risk factors for tinnitus in a young population. Otol Neurotol.

[3] McCormack A, Edmondson-Jones M, Fortnum H, Dawes P, Middleton H, Munro KJ, Moore DR (2014). The prevalence of tinnitus and the relationship with neuroticism in a middle-aged UK population. J Psychosom Res.

[4] Tyler RS, Baker LJ (1983). Difficulties experienced by tinnitus sufferers. J Speech Hear Disord.

[5] Andersson G, Edvinsson E (2008). Mixed feelings about living with tinnitus: A qualitative study. Audiol Med.

[6] Hoffman HJ, Reed GW, Snow JB (2004). Epidemiology of tinnitus. Tinnitus: Theory and Management. 1st ed.

[7] Department Of Health (2009). Provision of Services for Adults with Tinnitus: A Good Practice Guide.

[8] Gander PE, Hoare DJ, Collins L, Smith S, Hall DA (2011). Tinnitus referral pathways within the National Health Service in England: a survey of their perceived effectiveness among audiology staff. BMC Health Serv Res.

[9] Hoare DJ, Gander PE, Collins L, Smith S, Hall DA (2012). Management of tinnitus in English NHS audiology departments: an evaluation of current practice. J Eval Clin Pract.

[10] Hoare DJ, Broomhead E, Stockdale D, Kennedy V (2015). Equity and person-centeredness in provision of tinnitus services in UK National Health Service audiology departments. Eur J Pers Centered Heathcare.

[11] Williams C, Whitfield G (2001). Written and computer-based self-help treatments for depression. Br Med Bull.

[12] Griffiths F, Lindenmeyer A, Powell J, Lowe P, Thorogood M (2006). Why are health care interventions delivered over the internet? A systematic review of the published literature. J Med Internet Res.

[13] Jasper K, Weise C, Conrad I, Andersson G, Hiller W, Kleinstäuber M (2014). Internet-based guided self-help versus group cognitive behavioral therapy for chronic tinnitus: a randomized controlled trial. Psychother Psychosom.

[14] Nyenhuis N, Zastrutzki S, Weise C, Jäger B, Kröner-Herwig B (2013). The efficacy of minimal contact interventions for acute tinnitus: a randomised controlled study. Cogn Behav Ther.

[15] Andersson G, Strömgren T, Ström L, Lyttkens L (2002). Randomized controlled trial of internet-based cognitive behavior therapy for distress associated with tinnitus. Psychosom Med.

[16] Hesser H, Gustafsson T, Lundén C, Henrikson O, Fattahi K, Johnsson E, Zetterqvist WV, Carlbring P, Mäki-Torkko E, Kaldo V, Andersson G (2012). A randomized controlled trial of Internet-delivered cognitive behavior therapy and acceptance and commitment therapy in the treatment of tinnitus. J Consult Clin Psychol.

[17] Kaldo V, Haak T, Buhrman M, Alfonsson S, Larsen H, Andersson G (2013). Internet-based cognitive behaviour therapy for tinnitus patients delivered in a regular clinical setting: outcome and analysis of treatment dropout. Cogn Behav Ther.

[18] Greenwell K, Sereda M, Coulson N, El Refaie A, Hoare D (2016). A systematic review of techniques and effects of self-help interventions for tinnitus: Application of intervention coding methodology. Int J Audiol.

[19] Greenwell K, Featherstone D, Hoare Dj (2015). The application of intervention coding methodology to describe the Tinnitus E-Programme, an internet-delivered self-help intervention for tinnitus. Am J Audiol.

[20] Craig P, Dieppe P, Macintyre S, Michie S, Nazareth I, Petticrew M (2008). Developing and evaluating complex interventions: New guidance.

[21] Moore G, Audrey S, Barker M, Bonell C, Hardeman W, Moore L, O'Cathain A, Tinati T, Wight D, Baird J (2014). Process evaluation of complex interventions: UK Medical Research Council (MRC) guidance.

[22] Lichstein KL, Riedel BW, Grieve R (1994). Fair tests of clinical trials: A treatment implementation model. Advances in Behaviour Research and Therapy.

[23] Steckler A, Linnan L (2002). Process evaluation for public health interventions and research. 1st ed.

[24] Bellg AJ, Borrelli B, Resnick B, Hecht J, Minicucci DS, Ory M, Ogedegbe G, Orwig D, Ernst D, Czajkowski S, Treatment Fidelity Workgroup of the NIH Behavior Change Consortium (2004). Enhancing treatment fidelity in health behavior change studies: best practices and recommendations from the NIH Behavior Change Consortium. Health Psychol.

[25] Bradbury K, Watts S, Arden-Close E, Yardley L, Lewith G (2014). Developing digital interventions: a methodological guide. Evid Based Complement Alternat Med.

[26] Morrison LG, Hargood C, Lin SX, Dennison L, Joseph J, Hughes S, Michaelides DT, Johnston D, Johnston M, Michie S, Little P, Smith PW, Weal MJ, Yardley L (2014). Understanding usage of a hybrid website and smartphone app for weight management: a mixed-methods study. J Med Internet Res.

[27] Grant A, Treweek S, Dreischulte T, Foy R, Guthrie B (2013). Process evaluations for cluster-randomised trials of complex interventions: a proposed framework for design and reporting. Trials.

[28] Dennison L, Moss-Morris R, Yardley L, Kirby S, Chalder T (2013). Change and processes of change within interventions to promote adjustment to multiple sclerosis: learning from patient experiences. Psychol Health.

[29] Bendelin N, Hesser H, Dahl J, Carlbring P, Nelson KZ, Andersson G (2011). Experiences of guided Internet-based cognitive-behavioural treatment for depression: a qualitative study. BMC Psychiatry.

[30] Bennett GG, Glasgow RE (2009). The delivery of public health interventions via the Internet: actualizing their potential. Annu Rev Public Health.

[31] Glasgow RE, Lichtenstein E, Marcus AC (2003). Why don't we see more translation of health promotion research to practice? Rethinking the efficacy-to-effectiveness transition. Am J Public Health.

[32] Glasgow RE (2007). eHealth evaluation and dissemination research. Am J Prev Med.

[33] Yardley L, Morrison L, Bradbury K, Muller I (2015). The person-based approach to intervention development: application to digital health-related behavior change interventions. J Med Internet Res.

[34] Featherstone D Tinnitus E-Programme.

[35] Newman CW, Jacobson GP, Spitzer JB (1996). Development of the Tinnitus Handicap Inventory. Arch Otolaryngol Head Neck Surg.

[36] Creswell J, Plano Clark VL (2011). Designing and Conducting Mixed Methods Research. 2nd ed.

[37] Torrance H (2012). Triangulation, Respondent Validation, and Democratic Participation in Mixed Methods Research. Journal of Mixed Methods Research.

[38] Yardley L, Miller S, Teasdale E, Little P, Primit T (2011). Using mixed methods to design a web-based behavioural intervention to reduce transmission of colds and flu. J Health Psychol.

[39] Buchanan H, Coulson NS (2007). Accessing dental anxiety online support groups: an exploratory qualitative study of motives and experiences. Patient Educ Couns.

[40] Malik SH, Coulson NS (2008). Computer-mediated infertility support groups: an exploratory study of online experiences. Patient Educ Couns.

[41] Coulson NS (2013). How do online patient support communities affect the experience of inflammatory bowel disease? An online survey. JRSM Short Rep.

[42] Holbrey S, Coulson NS (2013). A qualitative investigation of the impact of peer to peer online support for women living with polycystic ovary syndrome. BMC Womens Health.

[43] Braun V, Clarke V (2006). Using thematic analysis in psychology. Qualitative Research in Psychology.

[44] Krefting L (1991). Rigor in qualitative research: the assessment of trustworthiness. Am J Occup Ther.

[45] Yardley L, Smith JA (2007). Demonstrating validity in qualitative psychology. Qualitative Psychology: A Practical Guide to Research Methods. 2nd ed.

[46] Charmaz K (2006). Constructing Grounded Theory: A Practical Guide to Qualitative Analysis. 1st ed.

[47] Joffe H, Yardley L, Marks DF, Yardley L (2004). Content and thematic analysis. Research Methods for Clinical and Health Psychology. 1st ed.

[48] Glaser BG, Strauss AL (1967). The discovery of grounded theory: Strategies for qualitative research. 1st ed.

[49] Polkinghorne DE (2005). Language and meaning: Data collection in qualitative research. Journal of Counseling Psychology.

[50] Coulson N (2015). Online Research Methods for Psychologists. 1st ed.

[51] Patton M (1990). Qualitative evaluation and research methods. 2nd ed.

[52] Webb TL, Joseph J, Yardley L, Michie S (2010). Using the internet to promote health behavior change: a systematic review and meta-analysis of the impact of theoretical basis, use of behavior change techniques, and mode of delivery on efficacy. J Med Internet Res.

[53] Geraghty AWA, Wood AM, Hyland ME (2010). Attrition from self-directed interventions: investigating the relationship between psychological predictors, intervention content and dropout from a body dissatisfaction intervention. Soc Sci Med.

[54] Nyenhuis N, Zastrutzki S, Jäger B, Kröner-Herwig B (2013). An internet-based cognitive-behavioural training for acute tinnitus: secondary analysis of acceptance in terms of satisfaction, trial attrition and non-usage attrition. Cogn Behav Ther.

