# Supplementary material for: Understanding User Reactions and Interactions With an Internet-Based Intervention for Tinnitus Self-Management: Mixed-Methods Process Evaluation Protocol
Source: JMIR Res Protoc. 2016 Mar 23;5(1):e49. doi: 10.2196/resprot.5008 (PMC4823589; doi:10.2196/resprot.5008)
Supplement: Multimedia Appendix 1 [file resprot_v5i1e49_app1.pdf]

Multimedia Appendix 1: Online survey for Study 1

Questions are consecutive, unless a different next question is specified in the brackets (e.g. [GO TO Q7]). Questions marked with an asterisk (*) are mandatory

TELL US ABOUT YOURSELF
**1. Which of the following describes how you think of yourself?***   • Male • Female • In another way (please say)
**2. What is your age?***   • 18-24 • 25-34 • 35-44 • 45-54 • 55-64 • 65-74 • 75+
**3. Which country do you live in?***   Drop-down box of countries
**4. Is English your first language?***   • Yes • No
**5. Do you have tinnitus?***   • Yes [GO TO Q8] • No, but I did have tinnitus previously [GO TO Q6] • No, I have never had tinnitus [GO TO Q7]
**6. Did you have tinnitus when you first visited the Tinnitus E-Programme website?***   • Yes [GO TO Q8] • No [GO TO Q7]
**7. What were your reasons for visiting the website?**   Comments box [GO TO Q10]
**8. Approximately how long is it since you first had tinnitus?***   • Less than 6 months • 6 months - 1 year • 2-5 years • 6-9 years • 10+ years
**9. Which of the following ways have you used to help you to manage your tinnitus?***   *You may be using these ways currently or have tried them previously but are no longer using them. Please select all that apply:*   • Seeking out information about tinnitus: ○ online,   ○ from books   ○ from other people with tinnitus   ○ from health professionals   • Sound therapy (e.g. hearing aids, sound therapy system, combination devices, noise generators)   • Relaxation   • Counselling or other psychological therapy (e.g. cognitive behavioural therapy)   • Tinnitus Retraining Therapy Medication (e.g. sleeping tablets, antidepressants)   • Taking part in **face-to-face** tinnitus support groups   • Taking part in **online** tinnitus support groups   • Contacting a charity or non-profit organisation (e.g. British Tinnitus Association, American Tinnitus Association)   • Other (please say)

**WHAT ARE YOUR EXPERIENCES OF USING THE TINNITUS E-PROGRAMME WEBSITE?**				
**10. How did you find out about the website?***<br>*Select one only:*<br>• Internet search engine (e.g. Google, bing)<br>• Health professional<br>• Word of mouth (e.g. someone with tinnitus)<br>• Researcher from NIHR Nottingham Hearing Biomedical Research Unit<br>• Other (please say)				
**11. Did you use the Tinnitus E-Programme?***<br>• Yes, all of it [GO TO Q12] • Yes, some of it [GO TO Q12] • No [GO TO Q13]				
**12. Why did you use the programme?**<br>Comments box [GO TO Q14 if person with tinnitus OR Q17 if person without tinnitus]				
**13. Why did you not use the programme?**<br>Comments box [GO TO Q37]				
**14. How do you use the programme now?***<br>*Select which option best describes you:*<br>• I have not used it since the first time I used it [GO TO Q15 then Q16]<br>• I still use at least some of the programme **regularly** [GO TO Q15 then Q18]<br>• I still use at least some of the programme **occasionally** [GO TO Q15 then Q18]				
**15. When was the last time you used any part of the programme?**<br>• In the last 6 months • 7 months - 1 year ago • More than 1 year ago				
**16. Do you think you will use the programme again in the future?***<br>• Yes • No [GO TO Q18]				
**[If person without tinnitus]**<br>**17. How did you use the programme?**<br>Comments box [GO TO Q44]				
Please click on the response that best describes you:	Strongly Agree	Agree	Disagree	Strongly Disagree
**18. The website was easy to use***				
**19. The website was visually attractive ***				
**20. The programme was a trustworthy source of support for tinnitus***				
**21. It was clear from the website which tasks I had to complete***				
**22. It was clear how much of my time I needed to spend on the programme***				
**23. The amount of time I needed to spend on the programme was achievable***				
**24. The order in which I needed to complete the tasks was clear***				

WHAT ARE YOUR EXPERIENCES OF USING THE INFORMATION LEAFLETS?

This section will ask about the **information leaflets** used in the programme.

[If used 'some of the programme']

25. Did you read the information leaflets?*

- Yes, all weeks [GO TO Q26]
- Yes, but only some of the leaflets [GO TO Q26]
- No, I did not read any of the information leaflets [GO TO Q30]
- Cannot remember [GO TO Q30]

Please click on the response that best describes you:	Strongly Agree	Agree	Disagree	Strongly Disagree	Did not use
**26. The information leaflets were easy to understand***					
**27. The information leaflets were relevant to me***					
**28. The information leaflets were helpful***					

29. Any other comments?

Comments box

WHAT ARE YOUR EXPERIENCES OF USING THE RELAXATION EXERCISES?

This section will ask about the **relaxation exercises** used in the programme.

[If used 'some of the programme']

30. Did you do any of the relaxation exercises?*

- Yes, all weeks [GO TO Q32]
- Yes, but only some of the exercises [GO TO Q32]
- No, I did not do any of the relaxation exercises [GO TO Q34]
- Cannot remember [GO TO Q34]

31. The recommendation is to practice the **Week 1 mind calming breathing exercise at least three times every day** and complete a **Week 2-5 30 minute recorded relaxation exercise every day** throughout the programme.

Do you feel this is achievable?*

- Yes
- No

32. The relaxation exercises were helpful*

Please click on the response that best describes you:

- Strongly Agree
- Agree
- Disagree
- Strongly Disagree
- Did not use

33. Any other comments?

Comments box

WHAT ARE YOUR EXPERIENCES OF USING THE TINNITUS HANDICAP INVENTORY?

This section will ask about the **Tinnitus Handicap Inventory**, a questionnaire used in the programme to find out what kind of problems tinnitus might be giving you.

[If used 'some of the programme']

34. Did you complete the Tinnitus Handicap Inventory?*

- Yes, at the beginning of the programme [GO TO Q35]
- Yes, at the end of the programme [GO TO Q35]
- Yes, at the beginning AND end of the programme [GO TO Q35]
- No, I did not complete the Tinnitus Handicap Inventory [GO TO Q37]
- Cannot remember [GO TO Q37]

35. The Tinnitus Handicap Inventory was helpful*

Please click on the response that best describes you:

- Strongly Agree
- Agree
- Disagree
- Strongly Disagree
- Did not use

36. Any other comments?

Comments box

WHAT ARE YOUR EXPERIENCES OF USING THE YAHOO! SUPPORT FORUM?

This section will ask about the **Yahoo! Support Forum**.

37. Did you join the Yahoo! Support Forum?*

- Yes [GO TO Q38]
- No [GO TO Q43]
- Cannot remember [GO TO Q44]

38. Did you read the posts of others?*

- Yes
- No
- Cannot remember

39. Did you post anything yourself?*

- Yes
- No
- Cannot remember

40. How do you use the forum now?*

- I do not use it anymore
- I use it **occasionally**
- I use it **regularly**

41. The forum was helpful*

Please click on the response that best describes you:

- Strongly Agree
- Agree
- Disagree
- Strongly Disagree

42. Any other comments?

Comments box [Users of the programme GO TO Q44; Users of the forum only GO TO Q47]

43. Why did you not to join the forum?

Comments box [GO TO Q44]

**WHAT EFFECT HAS THE TINNITUS E-PROGRAMME HAD ON YOU?**<br>This section will help us to understand the effect of the Tinnitus E-Programme on you. This includes the Yahoo! Support forum, if you used it.
**44. Did the programme help you?***<br>• Yes [GO TO Q45] • No [GO TO Q46]
**45. Please tell us how the programme helped you?**<br>Comments box [GO TO Q50]
**46. Please tell us why the programme did not help you?**<br>Comments box [GO TO Q50]

**WHAT EFFECT HAS THE YAHOO! SUPPORT FORUM HAD ON YOU?**<br>This section will help us to understand the effect of the Yahoo! Support Forum on you.
**47. Did the forum help you?***<br>• Yes [GO TO Q48] • No [GO TO Q49]
**48. Please tell us how the forum helped you?**<br>Comments box
**49. Please tell us why the forum did not help you?**<br>Comments box

**FINAL**
**50. Any other comments?**<br>Comments box
