# Supplementary material for: Understanding User Reactions and Interactions With an Internet-Based Intervention for Tinnitus Self-Management: Mixed-Methods Process Evaluation Protocol
Source: JMIR Res Protoc. 2016 Mar 23;5(1):e49. doi: 10.2196/resprot.5008 (PMC4823589; doi:10.2196/resprot.5008)
Supplement: Multimedia Appendix 2 [file resprot_v5i1e49_app2.pdf]

Multimedia Appendix 2: Interview Guide for Study 2

1. What were your initial impressions of the Tinnitus E-programme website?
2. What was your understanding of what would be expected of you throughout the programme?
3. Thinking back to before you started the programme. What did you personally hope to get out of it?
4. Can you talk me through how you used the programme across the 6 weeks?
5. Can you tell me about your experiences of using the information resources/relaxation exercises/Tinnitus Handicap Inventory/online discussion forum?
6. What did you like/dislike about the programme? Why?
7. Which components of the programme were the most helpful? Why?
8. Were there any components of the programme which you found were not helpful? Why?
9. In your opinion, has participating in the programme made any difference to how you manage your tinnitus? If so, how?
10. Do you think you will continue to use any other aspects of the programme after the 10 weeks? Which parts? If not, why?
11. In your opinion, how do you think the programme could be improved for future users?
